# A Comparison of Radical Perineal, Radical Retropubic, and Robot-Assisted Laparoscopic Prostatectomies in a Single Surgeon Series

**DOI:** 10.1155/2011/878323

**Published:** 2010-11-01

**Authors:** Moben Mirza, Kevin Art, Logan Wineland, Ossama Tawfik, J. Brantley Thrasher

**Affiliations:** ^1^Department of Urology-MS 3016, University of Kansas Medical Center, 3901 Rainbow Boulevard, Kansas City, KS 66160, USA; ^2^University of Kansas School of Medicine, Kansas City, KS 66160, USA; ^3^Department of Pathology, University of Kansas Medical Center, 3901 Rainbow Boulevard, Kansas City, KS 66160, USA

## Abstract

*Objective*. We sought to compare positive surgical margin rates (PSM), estimated blood loss (EBL), and quality of life outcomes (QOL) among perineal (RPP), retropubic (RRP), and robot-assisted laparoscopic (RALP) prostatectomies. *Methods*. Records from 463 consecutive men undergoing RPP (92), RRP (180), or RALP (191) for clinically localized prostate cancer were retrospectively reviewed. Age, percent tumor volume, Gleason score, stage, EBL, PSM, and QOL using the expanded prostate cancer index composite (EPIC) were compared. *Results*. PSM were similar when adjusted for stage, grade, and volume. EBL was significantly less in the RALP (189 ml) group compared to both RPP (475 ml) and RRP (999 ml) groups. When corrected for nerve sparing, there were no differences in erectile function and sexual function amongst the three groups. Urinary summary and pad usage scores showed no significant differences. *Conclusion*. RPP, RRP, and RALP offer similar surgical and QOL outcomes. RALP and RPP demonstrate less EBL compared to RRP.

## 1. Introduction

Radical prostatectomy remains the most commonly used treatment for clinically localized prostate cancer and can be performed by a variety of techniques. First performed by Young in 1904, the radical perineal prostatectomy (RPP) has been a proven technique for over 100 years. However, in the early 1980s, modifications to the radical retropubic prostatectomy (RRP) were introduced. RRP became the most popular surgical option and gained wider acceptance with the introduction of the nerve sparing technique by Walsh [1]. Large series comparing RRP with RPP have generally shown similar outcomes, except decreased blood loss associated with RPP [2, 3]. In more recent years, robot-assisted laparoscopic prostatectomy (RALP) and laparoscopic radical prostatectomy (LRP) have been introduced as minimally invasive techniques with associated benefits of shorter recovery periods, decreased postoperative pain, and smaller incisions [4].

There are multiple studies which have compared surgical outcomes between the different techniques including rates of positive surgical margin (PSM) among the different surgical modalities. Several studies have shown decreased PSM rates with RALP compared to RRP, yet others have demonstrated no advantage when RALP is used [5–8]. Regardless of their findings, these studies many times have inherent limitations introduced when data from multiple surgeons is compiled. This also creates potential bias in patient selection between the different surgical modalities which may impact results. Also, the popularity of RPP has been cyclical in nature since the introduction of RRP and RALP [2], a trend which may further complicate direct comparisons of the techniques. Although its effectiveness compared to RRP has been demonstrated, there is a paucity of data comparing RPP to RALP. In addition, there is lack of data comparing QOL outcomes between these groups. The purpose of our study was to evaluate the incidence and location of PSM among RPP, RRP, and RALP in 463 consecutive patients performed by a single surgeon (JBT) at one institution from March 2005 to February 2009 while controlling for differences in tumor biology, clinical, and pathological staging. We further sought to compare QOL outcomes between these groups using the EPIC questionnaire [9].

## 2. Materials and Methods

After exclusion of men receiving adjuvant therapy or undergoing salvage prostatectomy, the records of 463 men treated for clinically localized prostate cancer with RPP, RRP, and RALP from March 2005 to February 2009 were retrospectively reviewed. All radical prostatectomies (92 RPP, 180 RRP, and 191 RALP) were performed by a single surgeon (JBT) at one institution. All three surgeries were performed using standard techniques that have been previously described [3, 10, 11]. The decision of technique was made by the surgeon after thorough counseling with the patient regarding the risks and benefits of each procedure as well as both patient and tumor characteristics. RPP was favored in patients with morbid obesity, intra-abdominal mesh, renal transplant, or history of extensive abdominal or intraperitoneal surgeries. Otherwise, a discussion was undertaken with the patient where both RRP and RALP were offered. RALP was described to the patient as having the advantage of decreased EBL and earlier hospital discharge. 

Patient age, preoperative PSA level, Gleason score, final pathologic stage, percent tumor volume, risk classification, and PSM status were recorded. All pathologic specimens were reviewed by a single uropathologist (OWT) at one institution at the time of surgery. Percent tumor volume, PSM status and location, and pathological stage were noted based on the 1997 TNM classification [12]. PSM was defined as tumor present at the inked margin of the surgically resected prostate. PSM were classified based on their location: anterior, apical, posterolateral, and the bladder neck. Preoperative PSA, biopsy Gleason score, and 2002 AJCC clinical T-category were used to stratify groups by risk classification according to 2007 AUA Guidelines. 

EPIC questionnaires were obtained from the patients at all follow-up intervals. For the purpose of this study, questionnaires obtained between 12–18 months postoperatively were assessed. Complete and evaluable data were available on 177 patients, 35(38%) RPP, 46(26%) RRP, and 96(50%) RALP.

Statistical analysis was performed using SPSS 16 software (Chicago, IL). The means of parametric data were compared using *t*-test/ANOVA. Nominal data was examined using Chi-squared tests. *P* values of .05 or less were considered statistically significant.

## 3. Results

Clinicopathologic variables including EBL and length of stay were gathered for the patients and results are shown in Table 1. Preoperative PSA (ng/ml) was 5.64 in the RPP group, 8.89 in the RRP group, and 6.67 in the RALP group (*P* = .001). When grouped by Gleason sum, RALP cases were more likely to be Gleason sum ≤6 at 47.6% versus 28.3% for RPP and 21.1% for RRP (*P* = .013). When Gleason sum was 8 or greater, RPP and RRP were over four times more likely to be performed compared to RALP (*P* < .001). Patients undergoing RALP were more likely to have low-risk disease (44.5%) versus RPP (26.1%) and RRP (20.6%) patients. Patients with high-risk disease were approximately three times more likely to undergo RRP or RPP compared to RALP. Average percent tumor volumes were smallest in the RALP group (14.8%) compared to RPP (17.7%) and RRP (18%) groups (*P* < .001). Percent tumor volumes were then arbitrarily classified into groups of less than 10%, 10.1–20%, 20.1–30%, and greater than 30%. The number of RALP procedures performed was inversely proportional to percent tumor volume.

Overall, PSM occurred in 21.4% of RPP, 28.9% of RRP, and 13.6% of RALP (*P* = .007). As shown in Table 3, PSM incidence was lowest in low-risk patients undergoing all three modalities. Interestingly, high-risk patients were three times more likely to have PSM when undergoing RRP compared to those undergoing RALP (50% versus 15.4%), and twice as likely when undergoing RPP versus RALP. PSM rates in patients with T2 disease were 18%, 18%, and 11% for RPP, RRP, and RALP, respectively (*P* = .18). PSM rates in patients with T3 disease were 38%, 60%, and 37% for RPP, RRP, and RALP, respectively (*P* = .13).

PSM incidence rates based on percent tumor volume for each approach are shown in Table 2. Average percent tumor volume in PSM patients were similar among the different surgical approaches (24.1% in RPP, 26.7% in RRP, and 24.6% in RALP; *P* = .98). When grouped according to percent tumor volume, no statistically significant advantage was observed among the three modalities. Average EBL(ml) was 189 for RALP, 475 for RPP, and 999 for RRP (*P* < .001). EBL did not correlate with PSM incidence in any of the groups.

Incidence of PSM by margin location is shown in Table 4. Locations were classified as anterior, apex, bladder neck, or posterolateral. A few cases in each group contained more than one site of tumor involvement. In all three groups, the posterolateral margin was the most common site of tumor involvement. As expected, incidence of anterior margin involvement was highest in the RPP group (28.6%) compared to RRP (17.3%) and RALP (3.8%) groups. There was a trend for the RALP group having the smallest proportion of PSM at the bladder neck margin when compared to the other 2 techniques, although this difference did not reach statistical significance.

QOL scores are summarized in Table 5. There were no significant differences in the summary scores between the three groups for urinary, bowel, and hormonal scores. The sexual summary scores showed no significant differences between patients who underwent a nerve sparing procedure (Table 6). Average pad usage was 0.31, 0.23, and 0.42 for RPP, RRP, and RALP, respectively, with average EPIC score of 90, 92, and 86 for RPP, RRP, and RALP, respectively (rpp versus rrp *P* = .33, rpp versus ralp *P* = .25, rrp versus ralp *P* = .09). In patients undergoing nerve sparing procedure, the average scores for sexual function were 34, 35, and 38 for the RPP, RRP, and RALP groups, respectively, (rpp versus rrp *P* = .42, rpp versus ralp *P* = .26, rrp versus ralp *P* = .25). The average quality of erection scores in patient undergoing nerve spare procedure was 50, 57, and 60 for RPP, RRP, and RALP, respectively, (rpp versus rrp *P* = .28, rpp versus ralp *P* = .19, rrp versus ralp *P* = .36).

## 4. Discussion

In the present study, the PSM incidence was studied among RPP, RRP, and RALP surgical techniques in the treatment of clinically localized prostate cancer. All procedures were performed by a single surgeon (JBT) while pathological specimens were reviewed by a single pathologist at our institution (OWT). 

Tumors were stratified into low-, intermediate-, and high-risk categories based on the classification system outlined in 2007 AUA Guidelines [13]. Low-risk patients were more likely to undergo RALP, intermediate-risk RPP, and high-risk RRP, though these findings were not statistically significant. As expected, a higher PSM incidence was seen with increasing risk stratification for each group. When PSM incidence was analyzed for each risk classification, a statistically significant increased incidence was seen in high-risk patients but not for low and intermediate risk patients. Those undergoing RALP were less likely to have PSM. This conclusion supports the findings of 2 other studies comparing RALP and RRP. Ahlering and Laurila demonstrated no advantage in PSM rates between RRP and RALP in low- and intermediate-risk patients [7, 8]. Both of these studies were also single surgeon—a design which we believe strengthens their conclusions. 

 In contrast, studies by Smith and Tewari found decreased incidence of PSM in RALP patients compared to RRP [5, 6]. However, these conclusions were reached after analyzing results compiled from multiple surgeons. This raises the question of potential biases that are invariably introduced when more than one surgeon's outcomes are analyzed. Specifically, surgical skill and training as well as patient and technique selection may have impacted results. 

In our series, the potential impact of tumor volume on PSM rates among RPP, RRP, and RALP was studied. Chun et al. demonstrated that tumor volume is the most accurate univariate indicator of PSM [14]. Tumor volume is an important factor to consider when comparing PSM rates among surgical techniques as it is not accounted for in risk stratification profiles. In the present study, RALP patients had smaller tumor volumes (14.8%) compared to RPP (17.7%) and RRP (18.0%) patients (*P* = .056). However, when percent tumor volume was accounted for, no difference in PSM was seen. 

Pathologic staging showed a statistically significant increased likelihood of pT2 over pT3 disease in both RPP (77.2%) and RALP (87.7%) compared to RRP (67.2%) groups (*P* = .01). However, contrary to the findings of Smith and colleagues, there were no differences in PSM found among the groups for both pT2 and pT3 disease. These findings are in agreement with other studies [7, 15]. 

In nearly all studies to date, the apex is the most common site of tumor involvement in PSM patients [5, 8, 16]. It is accepted that this is due in large part to the less-defined prostatic capsule at the apex as well as the difficulty in dividing the apex in the RRP approach. However, the posterolateral margin was the most common site in our series. This may be due in part to a technique using sharp dissection of the apex during RRP rather than using the McDougal clamp as previously described [17]. We believe this modification, in addition to the improved apical visualization achieved during RALP, may account for this improvement in these 2 groups. The high proportion of posterolateral site PSM in all 3 groups is most likely a reflection of the difficulty in dividing the prostatic pedicle while preserving the neurovascular bundle. In general, the RPP approach grants improved access to the apex, though at the expense of anterior prostate exposure. Wieder reported the anterior prostate to be the most likely site of PSM in RPP [18]. Our series did find a greater likelihood of anterior PSM in the RPP group (28.6%) versus RRP (17.3%) and RALP (3.8%) groups (*P* = 0.07). However, the incidence of PSM at the posterolateral margin was equal to that of the anterior margin within the RPP group.

Since the popularity of RRP in the early 1980's and the more recent RALP, RPP has seen a decrease in number of procedures performed. However, it has consistently demonstrated similar oncologic outcomes to RRP [3, 19, 20]. In our series, the overall PSM rate in the RPP group (21.4%) was lower than that of the RRP (28.9%) group (*P* = .007). 

Urinary, sexual, bowel, and hormonal quality of life were not a function of the surgical technique. Median pad usage 12–18 months postoperatively in all groups was 0. Sexual summary, sexual function, and quality of erection were not affected by the surgical technique and showed no significant difference in patients who had undergone nerve sparing procedures. 

At our institution, RPP is the preferred method for radical prostatectomy in men with a history of significant prior abdominal surgery, renal transplantation, and/or morbid obesity. As current trends in the surgical management of clinically localized prostate cancer continue to emphasize minimally invasive techniques and shorter convalescence, we believe that RPP will continue to play a significant role in surgical options. Our results demonstrate that RPP is a valuable tool in the treatment of select men with clinically-localized prostate cancer.

Our current study has limitations. It is retrospective and nonrandomized in design. However to date, no randomized controlled study of the 3 modalities exists. Also, the decision of which particular surgical modality is reached by both surgeon as well as the patient, leaving room for possible bias. Response rate to EPIC questionnaire was a limitation. Quality of life data were not available at baseline (pre-op) or immediately post-op. We feel the strength of the study is that it is a large single-surgeon series with pathology reviewed by a single pathologist at our institution and the first to incorporate quality of life data in patients undergoing RPP.

## 5. Conclusion

In our series, RALP has a statistically significant lower overall rate of PSM compared to RPP and RRP. PSM also occurs less frequently in high-risk patients undergoing RALP. Tumor volume was overall less in the RALP group compared to RPP and RRP groups. When PSM incidence is compared among the groups based on tumor volume, no statistically significant difference in PSM incidence is observed. Quality of life outcomes are not affected by which surgical technique is employed. RPP continues to remain a proven effective alternative to RRP that offers similar oncologic outcomes with the benefits associated with a minimally invasive approach.

## Figures and Tables

**Table 1:** Patient Characteristics.

Variable		RPP (*n* = 92)	RRP (*n* = 180)	RALP (*n* = 191)	*P*-value
Age	Avg. (SD)	61.1(7.6)	61.7(6.8)	60.1(7.3)	0.12
Pre-op PSA (ng/mL)	Avg. (SD)	5.64(2.69)	8.89(9.44)	6.67(7.28)	0.001
Gleason score					
≤6	No. (%)	26(28.3)	38(21.1)	91(47.6)	<0.001
7		45(48.9)	82(45.6)	89(46.6)	0.87
8–10		21(22.8)	60(33.3)	11(5.5)	<0.001
Tumor volume (%)	Avg. (SD)	17.7(14.4)	18.0(15.8)	14.8(11.8)	0.056
≤10	No. (%)	40(43.5)	80(44.4)	103(53.9)	0.11
10.1 to 20		24(26.1)	44(24.4)	62(32.5)	0.21
20.1 to 30		14(15.2)	29(16.1)	27(14.1)	0.87
>30		14(15.2)	27(15)	15(7.9)	0.064
Risk classification					
Low	No. (%)	24(26.1)	37(20.6)	85(44.5)	<0.001
Intermediate		47(44.6)	83(46.1)	93(48.7)	0.73
High		21(22.8)	59(32.8)	13(6.8)	<0.001
Pathological stage					
T2	No. (%)	71(77.2)	131(72.8)	172(90)	<0.001
pT2a		18(19.6)	19(10.6)	27(0.14)	0.12
pT2b		2(2.2)	3(1.7)	5(2.6)	0.82
pT2c		51(55.4)	109(60.6)	140(73.3)	0.004
≥pT3a		21(22.8)	49(27.2)	19(9.9)	<0.001
EBL (mL)	Avg.	475	999.1	189.2	<0.001
Length of stay (days)	Avg.	1.29	2.28	1.23	<0.001

**Table 2:** PSM incidence based on % tumor volume among RPP, RRP, and RALP.

Tumor volume (%)		RPP (*n* = 21)	RRP (*n* = 52)	RALP (*n* = 26)	*P*-value
Less than 20	No. (%)	13(61.9)	26 (50)	13 (50)	0.63
20.1 to 30		3 (14.3)	13 (25)	6 (23.1)	0.62
Greater than 30		6 (28.6)	13 (25)	7 (26.9)	0.95

**Table 3:** PSM incidence based on risk classification among RPP, RRP, and RALP.

Risk classification		RPP (*n* = 21)	RRP (*n* = 52)	RALP (*n* = 26)	*P*-value
Low	No. (%)	2(9.5)	6(11.5)	5(19.2)	0.56
Intermediate		12(57.1)	20(38.4)	17(65.4)	0.06
High		7(33.3)	26(50)	4(15.4)	0.01

**Table 4:** Incidence of PSM by location among RPP, RRP, and RALP.

Location		RPP (*n* = 21)	RRP (*n* = 52)	RALP (*n* = 26)	*P*-value
Anterior	No. (%)	6(28.6)	9(17.3)	1(3.8)	0.07
Apex		4(19)	9(17.3)	5(19.2)	0.97
Bladder neck		6(28.6)	9(17.3)	2(7.7)	0.17
Posterolateral		6(28.6)	34(65.4)	20(76.9)	0.003

**Table 5:** Mean EPIC scores for urinary, bowel, hormonal parameters for RPP, RRP, and RALP.

Parameter	RPP	RRP	RALP	*P*-values		
	*N* = 35	*N* = 46	*N* = 96	rpp versus rrp	rpp versus ralp	rrp versus ralp
Urinary summary	88	86	83	0.24	0.24	0.10
Bowel summary	91	94	94	0.18	0.07	0.31
Hormonal summary	88	88	89	0.45	0.40	0.44
Pad usage	90	92	86	0.33	0.25	0.09

**Table 6:** Mean EPIC scores for sexual parameters for nerve sparing RPP, RRP, and RALP.

Parameter	RPP	RRP	RALP	*P*-values		
	*N* = 14	*N* = 33	*N* = 78	rpp versus rrp	rpp versus ralp	rrp versus ralp
Sexual summary	39	43	44	0.32	0.27	0.41
Sexual function	34	35	38	0.43	0.26	0.25
Quality of erection	50	57	60	0.28	0.19	0.36
